# Compound Heterogeneous Sickle Cell-B+ Thalassemia Incidentally Discovered Through Cytological Examination of a Fine-Needle Aspiration Specimen from an Aneurysmal Bone Cyst in a Young Child: A Case Report

**DOI:** 10.7759/cureus.33594

**Published:** 2023-01-10

**Authors:** Abdelrazak Meliti, Salmin Muftah, Daniyah Saleh, Nasrulla Habibullah

**Affiliations:** 1 Pathology and Laboratory Medicine, King Faisal Specialist Hospital and Research Centre, Jeddah, SAU

**Keywords:** hemoglobinopathies, sickle cell crisis, aneurysmal bone cyst, fine needle aspiration, sickle β thalassemia

## Abstract

Sickle cell beta-thalassemia (S/β) is a rare inherited variant of sickling disorders, usually occurring due to the inheritance of two abnormal genes, namely, the sickle cell gene, and the beta-thalassemia gene. There are two types of sickle cell β-thalassemia: (S/β+) and (S/β0), based on a decrease or complete absence of beta-globin synthesis, respectively. Skeletal complications, such as osteonecrosis, osteomyelitis, and septic arthritis, are well-known sequelae in sickle cell patients due to vaso-occlusive events. Nevertheless, the occurrence of aneurysmal bone cysts in patients with sickle cell hemoglobinopathy is an exceptional phenomenon. Herein, we report a case of a young boy who presented with nonspecific clinical symptoms over a few years. The patient was referred to our institution as a case of short stature with recurrent joint pain. A clinical workup was done and an aneurysmal bone cyst (ABC) of the left humerus was discovered radiologically with incidental detection of sickle cells through cytological evaluation of the cyst fluid. Further clinical investigation, including molecular and additional laboratory tests, confirmed the diagnosis of compound heterogeneous sickle cell-B+ thalassemia. Unfortunately, neither was the underlying pathology detected nor was the precise clinical diagnosis attained at the outside primary healthcare facility.

## Introduction

Sickle cell disease is an inherited autosomal recessive hemoglobinopathy that results from the substitution of β-chain (beta-chain) amino acid at valine residue, where glutamic acid is replaced by valine at the sixth amino acid position of the β-chain β6 (A3) glutamine valine [1].^ ^The disease has different genotypes with variable clinical symptoms and severity. Co-inheritance of the sickle cell gene and the beta-thalassemia gene gives rise to a condition known as compound heterozygous HbS/beta-thalassemia sickle cell variant. Compound heterozygosity represents 15% of patients with sickling disorders [2]. Based on a decrease or complete absence of beta-globin synthesis, two entities have been described, sickle cell β+ thalassemia and sickle cell β0 thalassemia, respectively [3]. Sickle cell β+ thalassemia can express variable clinical phenotypes, which are associated with the quantitative degree of hemoglobin A (HbA). The higher the level of HbA, the better the clinical outcome. Based on the relative concentration of HbA, S/b+-Thalassemia phenotypes can be classified into Type-I: 1-7% of HbA, Type-II: 7-14% of HbA, and Type-III: 14-25% of HbA [4]. A thorough clinical history, physical examination, complete blood count (CBC), and measurement of HbA2, HbS, and HbF levels can facilitate the correct diagnosis of HbS/beta-thalassemia. Also, high-performance liquid chromatography (HPLC) can be a useful adjunct to distinguish these disorders from sickle cell trait (AS), HbSC (Hemoglobin S/C), and other hemoglobinopathies [5]. We present a unique case of clinically undetected compound heterogeneous sickle cell b+-thalassemia with unusual clinical presentation and associated with an aneurysmal bone cyst (ABC) in an eight-year-old child initially discovered through cytological evaluation of the cyst fluid at our specialized tertiary hospital.

## Case presentation

**Figure 1:**
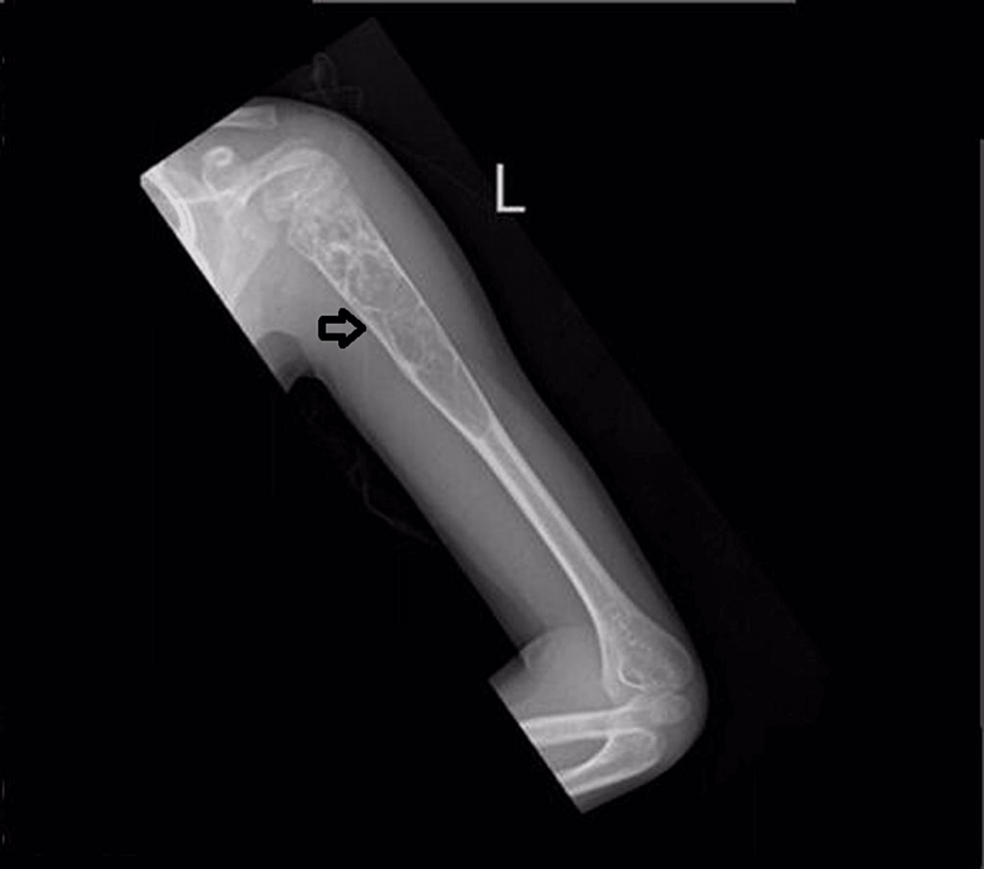
Plain X-ray of the left humerus Cystic lesion of the left upper humerus (arrow)

**Figure 2:**
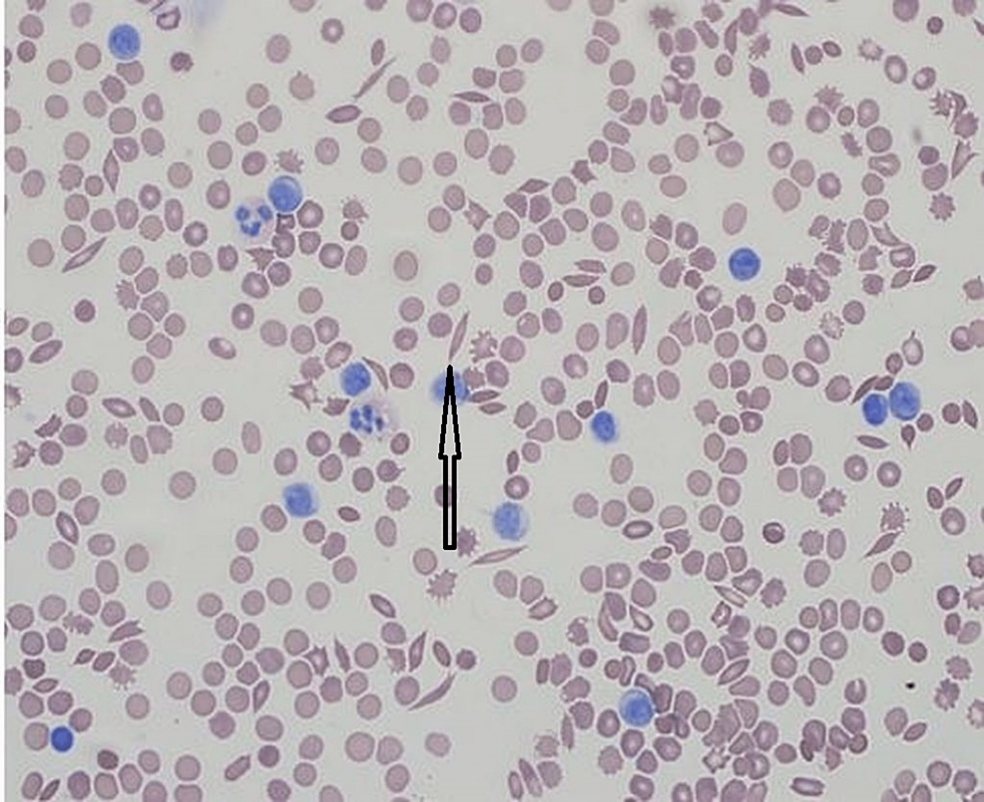
Diff-Quick cytology preparation (at 20x) Many sickle cells (arrow) in a lymphocyte-rich background rich admixed with a few histiocytes and rare plasma cells

An eight-year-old boy presented to our hospital for evaluation of short stature. He was not known to have any medical illness. A clinical history of episodes of joint pain over the last four years was given by the parents, provoked by moderate physical activities. He did not attend immediate clinical attention or was seen by a specialized tertiary hospital. He was investigated at his hometown primary healthcare facility, and no apparent underlying pathology was found to explain his clinical symptoms. Furthermore, his parents did not acknowledge any additional clinical information, laboratory workup, or provisional clinical diagnosis. The parents denied any history of blood transfusions. Besides, they did not acknowledge any prior history of hemoglobinopathies. However, a history of having a healthy sibling was given. On examination, the child appeared small for his age, pale but not jaundiced, with unremarkable conjunctiva. He was vitally stable. There was no splenomegaly and no lymphadenopathy. The X-ray images as part of investigations for recurrent joint pain revealed an incidental finding of a cystic lesion of the left upper humorous (Figure 1). The patient was seen by the orthopedic surgery team at our hospital and diagnosed clinically to have an ABC. Under general anesthesia, the patient underwent drainage and excision of the aneurysmal bone cyst followed by intralesional injection of cortisone and synthetic bone graft. The procedure went uneventfully. A fluid sample was obtained from the aneurysmal bone cyst and submitted for cytological examination. Microscopic examination reveals many sickle cells were identified in lymphocyte-rich background admixed with few histiocytes, and rare plasma cells (Figure 2). The cytological features were suggestive of sickle cell disease. As the above cytological findings were unexpected clinically, prompt communication with the orthopedic surgeon was established, and the hematology team was involved. Additional workup pursued at an outside facility was as follows: complete blood count (CBC), differential count, and Hb electrophoresis. CBC revealed mild anemia (Hb 9.9 mg/dl), microcytosis, and a normal iron profile. Repeat CBC and differential count at admission showed Hb 114 mg/dl with a normal differential count. Hb electrophoresis showed 63% HbS, 9.8% HbF, and 22% HbA with a mild elevation in HbA2 at 4.8%. Molecular analysis demonstrated a positive result for compound heterozygous c.92+ 6 T>C and heterozygous E6V mutations in the beta-globin HBB (hemoglobin subunit beta) gene in keeping with the above diagnosis. The patient has been followed up by pediatric hematology and endocrinology for almost four years, and he is doing well. His height at admission was 115.6 cm, and his most recent visit (six months ago) was 137.5 cm.

## Discussion

In 1963, Dr. Lehmman and colleagues were the first to describe the sickle cell gene in the Eastern part of Saudi Arabia [6]. Other hemoglobinopathies, such as HbS, were detected in other regions of the country. Additional studies have shown that the coexistence of the hemoglobin S gene with other hemoglobinopathies is not uncommon [7]. Clinically, sickle cell disease (SCD) could exhibit variable symptoms according to the degree of anemia; moreover, other sequelae, such as recurrent infections, ischemic crises, and stroke, are also documented. It is well-known that while some patients are transfusion-dependent, others may not require a single transfusion. Patients with severe disease who are transfusion-dependent may express variable clinical symptoms or may even present with complications such as growth retardation, bone defect, organ damage/ dysfunction alloimmunization, aneurysmal bone cyst, and other consequences secondary to frequent blood transfusions. On the other hand, patients with milder diseases do not usually experience such outcomes. Patients with sickle cell disease are likely to experience episodes of vaso-occlusive events affecting one or more organs with relapse and remission throughout their lifespan. Although skeletal involvement is considered a frequent problem in patients with sickle cell disease, it usually manifests as bone infarction/osteonecrosis secondary to a vaso-occlusive crisis, which ultimately requires hospitalization.

The pathogenesis, genetic alteration, and underlying etiology of the aneurysmal bone cyst are not well-established, yet many studies have suggested a possible linkage of secondary occurrence of aneurysmal bone cyst in patients with other primary bone and soft tissue tumors such as chondroblastoma, fibrous dysplasia, osteoblastoma, non-ossifying fibroma, chondromyxoid fibroma, hemangioma, eosinophilic granuloma, giant cell tumor, and others. Hence, aneurysmal bone cyst remains a descriptive terminology.

It is believed that an ABC usually occurs as a result of hemorrhagic degenerative events observed in patients with various bone and soft tissue lesions. The association between sickle cell disease and subsequent aneurysmal bone cyst formation is not well-established. However, some authors attribute the underlying vaso-occlusive crisis to the pathogenesis of aneurysmal bone cysts [8,9]. The association between sickle cell disease and subsequent aneurysmal bone cyst formation is also not well-established. However, some authors postulate that ischemic events due to a vaso-occlusive crisis lead to bone infarction, osteonecrosis, and ultimately, the formation of an ABC in patients with sickle cell disease [10].

Compound heterozygosity for HbS and b0- or b+-Thalassemia accounts for 15% of sickling disease. The majority of clinical symptoms of S/b-thalassemia depend on the presence or absence of HbA. S/b+ thalassemia individuals will have mild symptoms, whereas S/b0 thalassemia individuals will express a much more severe clinical course similar to patients with homogenous HbSS. In both types of S/b-Thalassemia, complete blood count (CBC) shows low mean corpuscular volume (MCV) and high-performance liquid chromatography (HPLC) demonstrates an increase in HbA2. The presence of HbA in double heterozygous HbS-b+ thalassemia may vary from <5% to 45%, and higher levels of HbA will dilute HbS and prevent cellular damage through inhibition of Hb polymerization. Furthermore, molecular studies have shown that among three mutations causing HbS- b+thalassemia type III, levels of HbA2, HbF, MCV, and mean corpuscular hemoglobin (MCH) were prevalent in the - 88 when compared to poly-A mutations. Clinically, HbS-b0thalassaemia and HbS-b+thalassemia type I were generally severe while HbS-b+thalassemia type III disease with - 88 mutation expressed a milder clinical course [11]. The current case is unique in that the patient presents with mild nonspecific clinical symptoms explained by the detection of coinheritance of sickle cell-b+ thalassemia, which has been accomplished through a high level of suspicion, careful cytomorphological examination and clinically confirmed with positive results for heterozygous mutations c.92+ 6 T>C and heterozygous E6V mutations in the beta-globin (HBB) gene. The T->C mutation at nt 6 of the first intron (IVS-I) reduces the efficiency of splicing at the 5' site. We hypothesize that in the presence of HbA: 22% and HbF: 9.8%, as in the present case, a milder form of the disease manifests itself clinically with nonspecific symptoms, which can mask the underlying pathology and be a diagnostic challenge in non-specialized primary healthcare facilities. We cannot emphasize enough the value of high clinical and pathological suspicion with the use of specific laboratory tests to early detect similar subclinical cases. Increasing the HbF level by prescribing hydroxyurea to symptomatic patients with HbS/b-thalassemia has been shown to improve the outcome by reducing the severity of the disease as reported in many studies [12]. At our institution, since 2001, more than 450 bone marrow transplantations were performed for patients with sickle cell disease. Bone marrow transplantation has significantly improved the outcome and the patient's quality of life. The therapy regimen includes hydroxyurea and folic acid. The patient has been followed up for almost four years, and he is doing well.

## Conclusions

Recognition of HbS in a more significant amount than HbA is the key to the correct diagnosis of sickle cell-b+ thalassemia and to differentiate it from the sickle cell trait. Both HbF and HbA appear to reduce the severity and improve the overall outcome of patients with sickle cell-b+ thalassemia in comparison with sickle cell- b0thalassaemia and homozygous sickle cell disease. Moreover, it is not uncommon for patients with sickle cell-b+ thalassemia to be clinically asymptomatic or present with unspecific signs and symptoms. Therefore, a high level of clinical suspicion and particular laboratory investigations should be observed for early detection of this disease to achieve a better quality of life. It is peculiar and sporadic to document the simultaneous occurrence of aneurysmal bone cysts in patients with sickle cell disease, an exciting phenomenon.
